# Knowledge, attitudes and practices on malaria transmission in Mamfene, KwaZulu-Natal Province, South Africa 2015

**DOI:** 10.1186/s12889-017-4583-2

**Published:** 2017-07-20

**Authors:** Pinky N. Manana, Lazarus Kuonza, Alfred Musekiwa, Hluphi D. Mpangane, Lizette L. Koekemoer

**Affiliations:** 1 South African Field Epidemiology Training Programme, National Institute for Communicable Diseases, Johannesburg, South Africa; 2 School of Health Systems and Public Health, Faculty of Health Sciences, University of Pretoria, Johannesburg, South Africa; 3 Division of Global Health Protection, U.S. Centers for Disease Control and Prevention (CDC), Pretoria, South Africa; 4 Wits Research Institute of Malaria, School of Pathology, Faculty of Health Sciences, University of the Witwatersrand, Johannesburg, South Africa; 5 Centre for Opportunistic, Tropical & Hospital Infections, National Institute for Communicable Diseases, Johannesburg, South Africa

**Keywords:** Malaria transmission, Knowledge, Attitude and practices, Sterile-insect technique, Survey, South Africa, KwaZulu-Natal

## Abstract

**Background:**

In South Africa malaria is endemic in Mpumalanga, Limpopo and the north–eastern areas of KwaZulu-Natal provinces. South Africa has set targets to eliminate malaria by 2018 and research into complementary vector control tools such as the Sterile Insect Technique (SIT) is ongoing. It is important to understand community perceptions regarding malaria transmission and control interventions to enable development of community awareness campaign messages appropriate to the needs of the community. We aimed to assess knowledge, attitudes, and practices regarding malaria transmission to inform a public awareness campaign for SIT in Jozini Local Municipality, Mamfene in KwaZulu-Natal province.

**Methods:**

We conducted a cross-sectional survey in three communities in Mamfene, KwaZulu-Natal during 2015. A structured field piloted questionnaire was administered to 400 randomly selected heads of households. Descriptive statistics were used to summarize data.

**Results:**

Of the 400 participants interviewed, 99% had heard about malaria and correctly associated it with mosquito bites. The sources of malaria information were the local health facility (53%), radio (16%) and community meetings (7%). Approximately 63% of the participants were able to identify three or four symptoms of malaria. The majority (76%) were confident that indoor residual spraying (IRS) kills mosquitoes and prevents infection. Bed nets were used by 2% of the participants. SIT knowledge was poor (9%), however 63% of the participants were supportive of mosquito releases for research purposes. The remaining 37% raised concerns and fears, including fear of the unknown and lack of information on the SIT.

**Conclusion:**

Appropriate knowledge, positive attitude and acceptable treatment-seeking behaviour for malaria were demonstrated by members of the community. Community involvement will be crucial in achieving success of the SIT and future studies should further investigate concerns raised by the community. The existing communication channels used by the malaria control program can be used; however additional channels should be investigated.

**Electronic supplementary material:**

The online version of this article (doi:10.1186/s12889-017-4583-2) contains supplementary material, which is available to authorized users.

## Background

Malaria is a public health challenge with approximately 214 million cases and 438,000 deaths occurring globally in 2015 [1, 2]. Africa accounts for over 80% of cases and 90% of deaths in the world with sub-Saharan Africa being the most affected malaria region [1, 2]. In South Africa (SA) the incidence of malaria was estimated at 20 cases per 100,000 population in 2015 [3]. The majority of the reported malaria cases in SA are currently attributed to *Plasmodium falciparum* infections, predominantly transmitted by *Anopheles arabiensis*, although *An. funestus* was implicated in the past [4]. The Malaria Control Programme (MCP) in SA focuses on vector control and case management [4, 5].

Vector control is primarily based on indoor residual spraying (IRS) [6, 7]. The IRS technique has been in operation effectively since the 1940s; however it has failed to completely eliminate malaria [7]. Limitations to the technique have been partly due to its limited suitability for indoor treatment and to insecticide resistance [8]. Insecticides that are currently used for IRS in SA include dicloro-diphenyl-trichloroethane (DDT) and the pyrethroid deltamethrin [4, 7]. South Africa is currently targeting elimination of malaria [1, 5]. However, additional strategies are needed to strengthen the current IRS based vector control intervention to achieve elimination. One such strategy is the Sterile Insect Technique (SIT) [9].

The SIT is an environmentally friendly, area-wide integrated pest management method [10]. Research conducted since the mid-1950s has shown SIT as an effective strategy in suppressing targeted insect populations, including mosquitoes [9]. The SIT process involves the inundative release of sterile males at high enough rates to cause a decline in a target wild vector population. It has been used successfully in the control of a number of insect pest species across the world: control of the Mediterranean fruit fly, *Ceratitis capitata* [11], eradication of tsetse fly *Glossina austeni* in the Island of Zanzibar [12] and eradication of the New World screwworm fly *Cochliomyia hominivorax* in the USA and Mexico [13, 14]. South Africa has been successfully using SIT to control the Mediterranean fruit fly in the Hex River Valley in the Western Cape since 1999 [15]. The feasibility of SIT as a malaria intervention tool is under investigation in several countries, including SA [16].

Prior studies conducted on malaria vector that circulate in the northern region of KwaZulu-Natal (KZN) province have reported increasing resistance to insecticides that are used for IRS [17, 18]. These findings have highlighted the need to implement additional/alternative vector control methods in order to sustain the reduction in malaria transmission in the population. Accordingly, the Mamfene area of northern KZN has been earmarked for a pilot study to assess the feasibility of using SIT for malaria vector control.

However, before a pilot programme is implemented, it is important to increase awareness about the programme in the target communities to ensure adequate cooperation and participation. Previous studies have demonstrated that directly engaging the community plays an important role in improving the acceptability and effectiveness of programmes aimed at reducing the transmission of malaria [9, 19]. Failure to consider the beliefs and perceptions of the community regarding aspects of the planned programmes may lead to negative attitudes or practices and contribute to failure to achieve the intended goals [9]. The SIT trials in the 1970s in New Delhi, India, to eradicate *Culex quinquefasciatus,* failed due to negative publicity in the community [9] and another trial in El Salvador, to eradicate *Anopheles albimanus,* was disrupted due to civil unrest [20]. Understanding community awareness of malaria transmission and concerns regarding control activities will allow research investigations to be modified to suit the needs of the community.

In order to provide community level baseline information and to inform public awareness campaigns specifically aimed at SIT we aimed to assess the knowledge, attitudes and practices of the Mamfene community in KZN regarding the transmission, prevention and treatment of malaria.

## Methods

### Study design and setting

**Fig. 1:**
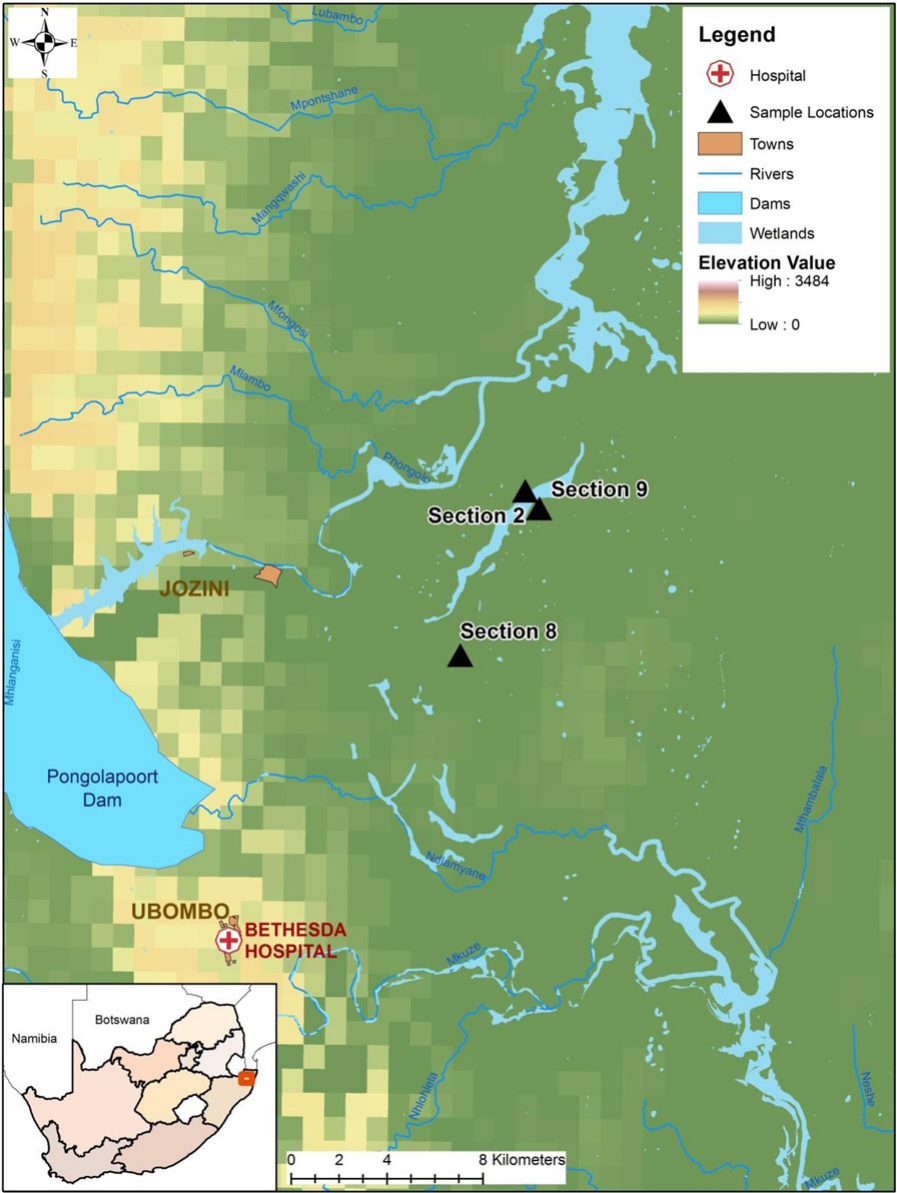
Map of Jozini Local Municipality showing the three sections in Mamfene, KwaZulu-Natal, South Africa (map adapted from Dandalo et al.: unpublished data)

We conducted a descriptive cross-sectional household survey during October 2015, in the Mamfene area of Jozini Local Municipality, which is situated in uMkhanyakude district of KwaZulu-Natal Province (Fig. 1). The district has five local municipalities including uMhlabuyalingana, Jozini, the Big Five False Bay, Hlabisa and Mtubatuba [21]. In 2011, the population in Jozini Local Municipality was estimated at 186,502 (29.8%) with ~39,000 households (in comparison to the other municipalities) [21] made up of the following age groups: 0–14 years (41.3%), 15–64 years (54.8%) and >65 years (3.9%). Over half (54%) of the population were females [22]. Mamfene comprises of 10 sections where Indoor Residual Spraying (IRS) is conducted annually and when a need has been identified. This study concentrated on three of the ten sections in Mamfene, Sections 2, 8 and 9. The three sections already serve as sentinel entomological surveillance sites for the KZN malaria control programme (MCP), and all three have also been selected for the assessment of the feasibility of implementing SIT. The population in the three sections was: section 2 (*n* = 2024); section 8 (*n* = 4592) and section 9 (*n* = 2167) [21–23].

### Data collection

Trained field workers administered a pre-piloted questionnaire that was translated into IsiZulu, the local language of the area, to respondents aged 18 years and older. The questionnaire was adapted from a tool that was previously used during a community KAP study on malaria in Swaziland in 2007 [24]. The final survey questionnaire was divided into five sections: demographics of the respondent; knowledge; attitudes; social practices and treatment-seeking behaviour related to malaria transmission and disease; and SIT. The questionnaire included closed-ended, partially closed and open-ended questions (the study questionnaire is available in supplementary file 1). The questionnaire was administered to 400 adult respondents. One key respondent, 18 years and older, was identified for each randomly selected household and preference was given to the head of the household when available.

### Sample size and sampling plan

The total population of the three sections was *N* = 8783. Since the proportions of knowledge, attitudes and practices regarding malaria transmission in this setting was unknown, a 50% proportion was assumed to ensure the maximum sample size. A sample size of *n* = 384 was calculated using Epi Info™ 7.1.4.0 assuming a population size of *N* = 9000, 50% prevalence, 95% confidence interval and 5% precision. We assumed a 96% response that resulted in a final sample size of 400 households.

The total number of households was allocated to the three sections proportional to population size, giving sample sizes of *n* = 90, *n* = 210 and *n* = 100 for sections 2, 8 and 9, respectively. Each section consists of six sentinel sites; all of which were used in the study. The sample size for each section was divided among the six sentinel sites such that each sentinel site contributed proportionate participants (15 study participants per site in section 2, 35 participants per site in section 8 and 17 participants per site in section 9).

### Data analysis

Data were captured on Epi Info™ 7.1.4.0 and exported to Excel. Descriptive statistical analysis was carried out using STATA Version 13. Measures of central tendency and dispersion were calculated for quantitative variables, and proportions were calculated for categorical variables. Frequency distribution tables and appropriate charts were displayed to show differences in the relative frequencies of variables. A Chi square test was used to determine whether there was a significant relationship between categorical variables. A *p* value <0.05 was considered statistically significant.

## Results

A total of 400 questionnaires were completed, with no refusals and 100% of questions completed. The majority of the participants were females 71% (*n* = 285). Ages of the participants ranged between 18 and 90 years old, with a mean age (SD) of 42 (±16.5) years. Heads of household accounted for 37% (*n* = 149) of the respondents. The majority of the respondents, 71% (*n* = 286) were unemployed and most had attained at least primary level education, 71% (*n* = 284). In addition, 12% (*n* = 47) of the respondents reported that at least one family member had parasitological confirmed (by Rapid Diagnostic Test) malaria in the preceding year (2014) (Table 1).

**Table 1:** Demographic characteristics and malaria history among participants in Mamfene, Jozini, KwaZulu-Natal, South Africa 2015

Characteristics	*n* = 400	Percent
Gender		
Male	115	28.8
Female	285	71.2
Age (in years)		
18–35	163	40.8
36–50	111	27.7
51–65	78	19.5
> 65	45	12.0
Family position		
Head of household	149	37.2
Wife	112	28.0
Children	129	32.3
Other	10	2.5
Highest level of education attained		
No schooling	116	29.0
Primary level	131	32.7
Secondary level	144	36.1
Tertiary level	9	2.2
Employment status		
Employed	21	5.3
Unemployed	286	71.5
Housewife	15	3.8
Pensioner	71	17.7
Seasonal worker	1	0.2
Student	6	1.5
Family history of malaria in 2014^a^		
Had malaria infection	47	11.8
No malaria infection	352	88.0
Do not know	1	0.2

^a^At least one member of the household suffered from malaria infection during 2014

### Knowledge of malaria transmission

**Fig. 2:**
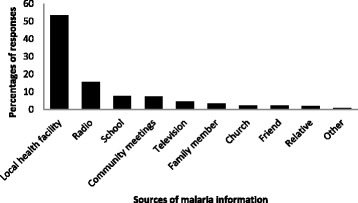
Sources of malaria information as identified by the participants in Mamfene Jozini, KwaZulu-Natal, South Africa, 2015

Of the 400 participants interviewed, 99% (95% CI: 97.8–99.8%) had heard about malaria before and correctly associated malaria with mosquito bites. All participants who had heard about malaria further reported that malaria can kill if it is not treated. Only 32% (95% CI: 27.4–36.8%) associated the female mosquito as the carrier of infection. The most commonly identified source of malaria information were the local health facility 53% (95% CI: 48.2–58.2%), radio 15% (95% CI: 12.0–19.4%) and community meetings 7% (95% CI: 4.9–10.2%), with community health promoters and pamphlet distribution contributing only less than 1% (Fig. 2). Approximately 59% (95% CI: 54.5–64.3%) reported not to have enough information on malaria and were keen to know more.

**Fig. 3:**
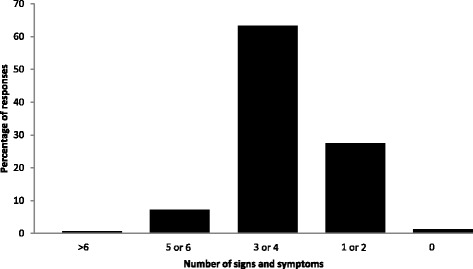
Number of malaria signs and symptoms identified by the participants in Mamfene, Jozini, KwaZulu-Natal, South Africa, 2015

The most frequently reported signs and symptoms of malaria included headaches (78%), fever (54%), feeling cold (60%) and vomiting (30%). Approximately 63% of the participants were able to correctly identify three or four of the symptoms (Fig. 3). Only 9 % (*n* = 36) of the participants had heard about the SIT. However, the majority of the participants, 63% (95% CI: 57.8–67.5%), had a positive response with regards to releasing mosquitoes into the environment for research purposes, after the study team described the purpose and practice of SIT to them (Table 2).

**Table 2:** Participants knowledge of the sterile insect technique (SIT) in Mamfene Jozini, KwaZulu-Natal, South Africa 2015

Knowledge questions		
Participants heard about SIT before	*n* = 400	%
Yes	36	9.0
No	364	91.0
Where did participants hear about SIT	*n* = 36	
Community meetings	30	83.3
Health care facility	1	2.8
Neighbour	3	8.3
Church	2	5.6
Participants’ understanding on SIT	*n* = 36	
To assist in elimination of malaria	33	91.0
Do not know	3	9.9
Participants’ opinion on mosquito release	*n* = 400	
Those who are for it	251	63.0
Those who are against it	149	37.0

The 18–50 years age group were more in support of the SIT compared to the 50+ years age group (66% [182/274] versus 55% [69/126], *p* = 0.025). The other members of the household (wife, children or other) were more in support of the SIT compared to the heads of the household (71% [177/251] versus 50% [74/149], *p* < 0.001). The most reported reason for not supporting SIT by the remaining 37% (*n* = 146) of participants was the fear that the mosquitoes would bite and cause illness. The second reported reason was that they did not understand the concept of SIT.

### Attitudes toward malaria

In terms of prevention, 95% (95% CI: 92.9–97.3%) of participants reported that malaria can be prevented through various preventive activities, 2% (*n* = 6) did not know, and 3% (*n* = 12) reported it cannot be prevented. Approximately 49% (95% CI: 44.2–54.2%) reported to be using personal protective products/substances currently in their households, such as mosquito coils (55%), burning cow dung (17%), mosquito repellent (14%), insecticide (7%), burning tissues (5%) and 2% noted use of bed nets, burning plants, closing windows, garlic and Vaseline gel. Approximately 75% (95% CI: 70.4–79.1%) reported that protection against malaria was attained by indoor residual spraying (IRS). The majority (76%) were confident that IRS kills mosquitoes and 74% were also confident that it prevents infection. At least 98% reported that it is important to allow IRS operators to spray inside their houses. Of the 400 participants, 42% reported that malaria is still a problem in the area, 53% reported that it is not a problem, and 5% did not know.

### Malaria practices and treatment seeking behaviour

Of the 400 participants, only nine (2%) reported having bed nets in the households. We found that of these nine with bed nets, five were used by the mothers, three by the fathers, and one by the sister in the households. Only one bed net was not used as a result of wear-and-tear. Approximately 99% of the houses were being sprayed on an annual basis, with the other remaining 1% reporting that houses were new in the area. About two thirds (66%) of the houses were last sprayed in 2014. The reason given for non-spraying was mainly due to houses being locked and the homeowner not being available during the spraying schedule. The majority (99%) reported that they would seek treatment from a health care facility if anyone in the household were to develop signs and symptoms suggestive of malaria. Approximately 63% reported treatment seeking within 24 h of experiencing symptoms.

## Discussion

In SA, this is the first study that has been carried out to provide baseline information on malaria related knowledge, attitude and practices at community level, as the first step to introduce SIT.

Adequate knowledge, encouraging attitudes, and suitable treatment seeking behaviour were demonstrated by the participants in the community of Mamfene. However, few people had heard about SIT and older respondents raised concerns about SIT as a malaria control strategy. The lack of awareness and concerns should be addressed as part of an SIT communication initiative.

The community’s knowledge on malaria was generally good; however, more than half of the participants also indicated that they would like to acquire more knowledge regarding malaria. The most identified sources of information by the participants were the local health facility, radio and community meetings. Participants felt that community health promoters contributed very little information about malaria and information leaflets or booklets were not distributed adequately. We found that almost all of the participants knew about malaria and correctly associated malaria with mosquito bites, congruent with the findings of studies in Mpumalanga province [25] and neighbouring Swaziland [24]. Only about a third of the participants correctly identified the female mosquito as the carrier of infection. This will be an important point to consider when developing community health awareness messages for the SIT project, given that only non-biting male mosquitoes are released into the environment during SIT.

More than half of the participants were able to identify the most common signs and symptoms of which were headache, fever, feeling cold and vomiting. This is an important result as these are the early symptoms experienced by individuals who are infected with malaria. It also matches the WHO target for knowledge [1] and is also in accordance with findings in other studies in endemic settings [24–26].

A positive attitude regarding malaria control was demonstrated. However, approximately 22% of the participants reported the use of unproven methods of malaria control, such as burning cow dung, that are unlikely to provide effective protection against malaria transmission. This could have been a result of individual perceptions driven by previous experiences. Additional scope of research for improving control measures has to be investigated and encouraging the community to use available conventional methods is vital. In addition, half of the participants reported not taking any personal protective measures to guard against malaria infection. This could be due to the fact that most people are dependent on the IRS programme or information claiming that malaria is no longer a threat in their area (reported prevalence of malaria in KZN is <1 case per 1000 population) [3]. According to a study in Mpumalanga province in 2008 on knowledge and practices towards malaria, approximately a quarter of the participants reported not to use any personal protection against malaria [25].

The majority of participants reported that their households are sprayed on an annual basis and two-thirds noted that they were last sprayed in 2014. This finding is below what is expected according to the WHO guideline on IRS coverage which recommends a minimum of 80% within the targeted communities [6]. Although IRS coverage was less than expected, the majority of participants were confident that spraying kills mosquitoes and prevents infection. While bed nets have never been part of the vector control intervention in SA, the Department of Health encourages use of bed nets for personal protection. However, use of bed nets was low with only 2% of the households reported having bed nets, possibly because SA does not have a bed nets distribution programme. The last time Mamfene was supplied with bed nets was in 2000 by the Medical Research Council of South Africa (MRC) during an awareness campaign [27].

The majority of participants reported seeking treatment at a local health facility. This could be due to the perceived quality and accessibility to health care facilities in KZN. More services are now accessible and testing is also done on site in local facilities, including active case finding in the province [27]. Approximately two-thirds of the participants in the study reported that they sought treatment within 24 h after having experienced any signs and/or symptoms of malaria. No mention of traditional healers was reported.

More than half of the participants were in support of the SIT strategy. Participants aged 50 years and younger and other members of the household were more in support of the SIT. Participants raised concerns of being afraid of mosquitoes and fearing the unknown. These are concerns that should be addressed during awareness campaigns.

A limitation of the study was that participants’ migration history was not considered. Given the degree of cross-border movement between Swaziland, Mozambique and KZN this could have an impact on the participants who reported that malaria was still a problem and not having enough information on malaria. According to Moonasar et al. 2013, it was reported that approximately 50% of infections reported in KwaZulu-Natal and approximately 80% reported in Mpumalanga are imported malaria cases mostly from Mozambique [5]. Additionally, the three sites from which participants were sampled from were conveniently selected, thereby limiting the representativeness and generalizability of the findings. However, participants were randomly selected and the study utilized a statistically calculated sample size giving sufficient power for the study.

## Conclusion

We reported findings of the knowledge, attitudes and practices of the Mamfene community, an endemic malaria province in South Africa. Some key findings were that the community had reasonable knowledge of malaria transmission, but showed an interest in learning even more. Furthermore, the community were in support of passive vector control strategies such as IRS, and SIT will therefore be a supplementary passive vector control intervention. Although SIT was largely supported, a substantial proportion of the community will require more information from communication platforms identified here. This communication can be channelled through the health facility staff already involved in providing basic malaria and malaria control messages without the need for additional resources apart from additional training on SIT to ensure the message to the community is accurate and appropriate. The SIT campaign should also communicate the technology to the community to cover all age groups and to translate the scientific terminology to simple language.

## Additional files

Additional file 1: KZN KAP_Questionnaire1. (XLSX 189 kb)

Additional file 2: KZN KAP Data. (DOCX 38 kb)
