# Supplementary material for: Knowledge, attitudes and practices on malaria transmission in Mamfene, KwaZulu-Natal Province, South Africa 2015
Source: BMC Public Health. 2017 Jul 20;18:41. doi: 10.1186/s12889-017-4583-2 (PMC5520288; doi:10.1186/s12889-017-4583-2)
Supplement: Supplementary file 2 — KZN KAP Data. (DOCX 38 kb) [file 12889_2017_4583_MOESM2_ESM.docx]

# Questionnaire


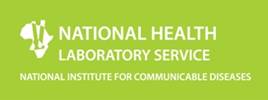

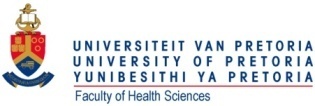


Title: Malaria transmission knowledge, attitude and practices in Mamfene Jozini district in KZN 2015

**Date of interview**: ______/_____/2015

**Unique household number**: ____________________ (sought advice on coding system)

**Name of interviewer**: _________________________________

| **Start time** |  | **Finish time** |  |
| --- | --- | --- | --- |

| **Malaria transmission knowledge, attitude and practices household survey. Baseline 2015.MamfeneJoziniKwaZulu Natal** | | |
| --- | --- | --- |
| **Instruction**:  I am conducting this study towards completion of my Master degree in Public Health as an educational requirement. Please give me a few minutes of your time to complete this questionnaire. The questions that you will be asked include information on knowledge, attitude and practices regarding the malaria disease and preventative measures thereof. Your may interrupt the interview at anytime and if there is anything that is unclear, feel free to ask me. Please note that your contribution in this study is highly appreciated. There is no wrong and right answer. The information you provide will be treated confidentially and with dignity. Please do not put your name on the form. Answer the questions as honest as possible as that will allow us to have a clear understanding of what is known and unknown, and in turn, plan relevantly according to the needs of this community. | | |
| SECTION 1: DEMOGRAPHIC CHARACTERISTICS | | |
| Questions | Coding categories | Answer code |
| 1.1 Age | ________years  (age in current year) |  |
| 1.2 Gender | 1=Male  2=Female |  |
| 1.3 What is your position in the family | 1=Head of the House  2=Husband  3=Wife  4=Daughter/Son  5=Brother/Sister  6=Friend  7=Other_________________ |  |
| 1.4 Highest level of education attained | 1=No schooling  2=Primary level  3=Secondary level  4=Tertiary level (degree or diploma)  5=Postgraduate  6=Other_______________ |  |
| 1.5 Employment status | 1=Student  2=Housewife  3=Unemployed  4=Employed  5=Pensioner  6=Seasonal worker |  |
| 1.6 If employed, please indicate type of employment | 1=Farm worker  2=Domestic worker  3=Non – skilled office  4=Skilled office  5=Other____________________ |  |
| 1.7 Has anyone in the family suffered from malaria in the past year (2014)? | 1=Yes  2=No  3=Don’t know |  |
| 1.8 Main material of the walls in the household | 1=Cane  2=Stones with mud  3=Cement with stone  4=Clay blocks  5=Sticks and mud  6=Cement bricks  7=Other______________________ |  |
| 1.9 Main material of the roof | 1=Grass  2=Tiles  3=Asbestos  4=Rustic mat  5=Reed  6=Other__________________ |  |
| 1.10 Type of toilet facility the household is using | 1=Flushing toilet  2=Traditional Pit toilet  3=Ventilated improved pit toilet  4=Bucket toilet  5=No facility/ bush  6=Other__________________ |  |
| 1.11 Main source of drinking water for the family | 1=Running tap water in dwelling  2=Public well  3=Surface water (river, dam, pond)  4=Piped water into the plot  5=Rainwater  6=Other_____________________ |  |
| 1.12 Does this household own any of the following?  (Multiple responses allowed) | 1=Radio  2=Television  3=Car  4=Mobile phone  5=Electricity  6=Tractor  7=Bicycle |  |
| 1.13 What industry do you work in? | 1=Construction  2=Shop  3=Farming  4=Mining  5=Public sector  6=Private sector  7=Transport  8=Non – governmental organisation  9=Other___________________ |  |
| SECTION 2: GENERAL KNOWLEDGE ABOUT MALARIA | | |
| 2.1 Have you heard about Malaria before? | 1=Yes  2=No |  |
| 2.2 If you answered yes to question 2.1, where did you hear about Malaria? | 1=TV  2=Radio  3=Newspaper  4=Friend  5=Family member  6=Relative  7=Local health facility  8=School  9=Church  10=Work  11=Community meetings  12=Pamphlets  13=Other_________________ |  |
| 2.3 What are the main signs and symptoms of Malaria?  (Multiple responses allowed) | 1=Fever  2=Headache  3=Joint pains  4=Feeling cold  5=Nausea and vomiting  6=Diarrhoea  7=Dizziness  8=Loss of appetite  9=Body weakness  10=Delirium  11=Other___________________ |  |
| 2.4 In your opinion, what causes Malaria?  (Multiple responses allowed) | 1=Mosquito bite  2=Witchcraft  3=Drinking dirty water  4=Cold weather  5=Unhygienic environment  6=Close contact with a malaria sufferer  7=Other____________________ |  |
| 2.5 How can someone protect himself/herself against Malaria?  (Multiple responses allowed) | 1=Keep house surrounding clean  2=Eat garlic  3=Use mosquito repellent  4=Avoid mosquito bite  5=Take preventative medication  6=Use mosquito coils  7=Sleep under mosquito nets  8=Avoid drinking dirty water  9=Keep warm at all times  10=Avoid contact with malaria sufferer  11=Allow spray operators to spray your house  12=Other__________________ |  |
| 2.6 Do you think malaria can kill you, if it is untreated? | 1=Yes  2=No  3=Don’t know |  |
| 2.7 Do you think you have enough information on malaria? | 1=Yes  2=No  3=Don’t know |  |
| 2.8 Do you think a mosquito causes malaria? | 1=Yes  2=No |  |
| 2.9 If you answered yes to question 2.8, is it a male or female mosquito that causes malaria? | 1=Male  2=Female  3=Both  4=Don’t know |  |
| 2.10 If you answered no to question 2.7, what information would you like to get? | 1=Information on transmission  2=Information on prevention  3=Information on control  4=Information on treatment  5=Information on signs and symptoms  6=Any information  7=Other______________________  8=Don’t know |  |
| 2.11 How would you like this information to be communicated to you? | 1=TV  2=Radio  3=Newspaper  4=Church  5=Community meetings  6=Pamphlet and posters  7=Health facility  8=Work place  9=Other__________________  10=Don’t know |  |
| SECTION 3: ATTITUDE ABOUT MALARIA | | |
| 3.1 Do you think malaria can be prevented? | 1=Yes  2=No  3=Don’t know |  |
| 3.2 If you answered yes to question 3.1, how can it be prevented? ________________________ | | |
| 3.3 If malaria is not treated, can it kill you? | 1=Yes  2=No  3=Don’t know |  |
| 3.4 Do you think malaria is still a problem in your area? | 1=Yes  2=No  3=Don’t know |  |
| 3.5 How confident are you that spraying the inner walls of the house with insecticide, known as indoor residual spraying (IRS) kills the mosquito? | 1=Very confident  2=A little confident  3=Not at all confident  4=Don’t know |  |
| 3.6 How confident are you that the indoor residual spraying with insecticide can prevent malaria infection? | 1=Very confident  2=A little confident  3=Not at all confident  4=Don’t know |  |
| 3.7 Do you think it is important to seek treatment when you suspect that you are infected with malaria? | 1=Yes  2=No  3=Don’t know |  |
| 3.8 Do you think is important to allow spray operators to spray your house structures? | 1=Yes  2=No  3=Don’t know |  |
| 3.9 Does anyone in the household use any personal protective items to help prevent malaria infection? | 1=Yes  2=No  3=Don’t know |  |
| 3.10 If you answered yes to question 3.9, which items? _____________________________ | | |
| 3.11 How do you feel about mosquitoes being released in your area for research purposes?  _________________________________________________________________________ | | |
| SECTION 4: PRACTICES AND TREATMENT – SEEKING BEHAVIOUR | | |
| 4.1 What personal protective measures do you use to guard against malaria infection? | 1=Use repellents  2=Use mosquito coils  3=Use doom  4=Burn cow dung  5=Use mosquito nets  6=Close windows and doors  7=Do nothing  8=Other________________ |  |
| 4.2 Does the household have bed nets? | 1=Yes  2=No  3=Don’t know |  |
| 4.3 If you answered yes to question 4.2, who uses the bed net? | 1=Father  2=Mother  3=Children over 5 years  4=Children under 5 years  5=Other________________ |  |
| 4.4 Where available are the nets being used? | 1= Yes  2=No  3=Don’t know |  |
| 4.5 If you answered no to question 4.4, why not? _________________________________ | | |
| 4.6 Is this household being sprayed by the department of health against mosquitoes? | 1=Yes  2=No  3=Don’t |  |
| 4.7 If you answered yes to question 4.6, how often? ___________________ | | |
| 4.8 When was the last time? ____________________________ | | |
| 4.9 If you answered no to question 4.6, why not? ___________________________ | | |
| 4.10 If you or anyone in the household presented with signs and symptoms of malaria where would you seek treatment? | 1=Health facility  2=Traditional healer  3=Pharmacy  4=Nowhere  5=Self treat  6=Other_________________ |  |
| 4.11 How soon after suspecting will you seek treatment? | 1=One day (within 24hrs)  2=2-3 days  3=4-6 days  4=7 days and more  5=Other______________________ |  |
| SECTION 5: KNOWLEGDE ON SIT (Sterile Insect Technique) | | |
| 5.1 Have you heard about the sterile insect technique (SIT)? | 1=Yes  2=No  3= Don’t know |  |
| 5.2 If you answered yes to question 5.1, where did you hear about it? ________________ | | |
| 5.3 In your own words, what do you understand about SIT? _______________________ Thank you very much.We have come to the end of our interview. Your time, honest opinions and valuable contributions are highly appreciated. Do you have any questions for me?____________________________________________________________________________________________________________________________________________________________Finish time:_______________________Any important observations (if any) | | |
